# Resveratrol counteracts bone loss via mitofilin-mediated osteogenic improvement of mesenchymal stem cells in senescence-accelerated mice: Erratum

**DOI:** 10.7150/thno.74941

**Published:** 2022-07-08

**Authors:** Ya-Jie Lv, Yi Yang, Bing-Dong Sui, Cheng-Hu Hu, Pan Zhao, Li Liao, Ji Chen, Li-Qiang Zhang, Tong-Tao Yang, Shao-Feng Zhang, Yan Jin

**Affiliations:** 1 State Key Laboratory of Military Stomatology, Center for Tissue Engineering, School of Stomatology, Fourth Military Medical University, Xi'an, Shaanxi 710032, China.; 2 Xi'an Institute of Tissue Engineering and Regenerative Medicine, Xi'an, Shaanxi 710032, China.; 3 State Key Laboratory of Military Stomatology, Department of Prosthodontics, School of Stomatology, Fourth Military Medical University, Xi'an, Shaanxi 710032, China.; 4 Department of Dermatology, Tangdu Hospital, Fourth Military Medical University, Xi'an, Shannxi, 710069, China.; 5 Department of Orthopaedics, Tangdu Hospital, Fourth Military Medical University, Xi'an, Shannxi, 710069, China.

The authors apologize that the original version of the above article contains errors that need to be corrected. Incorrect images for ALP staining in Figure 5K were used in figure assembly. The authors apologize for any inconvenience these errors may have caused. Luckily the correction does not affect the conclusions of the above paper. The corrected Figure 5K appears below.

## Figures and Tables

**Figure 5:**
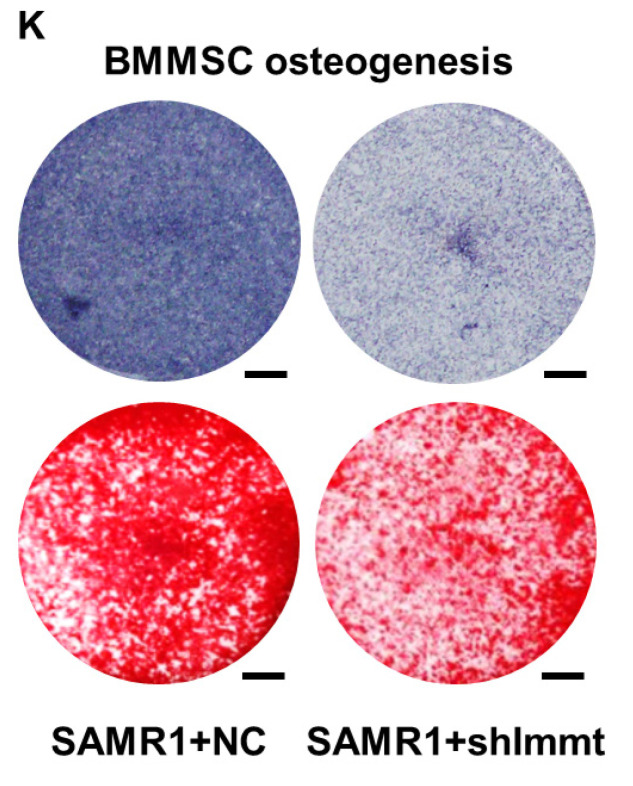
Corrected figure for original Figure 5K.

